# Dr. Kiyoshi Shiga (1871–1957): Outstanding Bacteriologist Who Discovered Dysentery Bacillus and Contributed Immensely to Public Health in Japan

**DOI:** 10.7759/cureus.71881

**Published:** 2024-10-19

**Authors:** Nobuo Okui

**Affiliations:** 1 Urogynecology, Yokosuka Urogynecology and Urology Clinic, Kanagawa, JPN; 2 Dentistry, Kanagawa Dental University, Kanagawa, JPN

**Keywords:** bacteriology, dysentery bacillus, historical vignette, infectious disease, kiyoshi shiga, public health

## Abstract

Kiyoshi Shiga (1871-1957) was a renowned Japanese bacteriologist best known for discovering the dysentery bacillus. Born in Sendai during the Meiji Restoration, Shiga graduated from Tokyo Imperial University's School (later University of Tokyo) of Medicine and began microbiological research under Shibasaburo Kitasato. In 1897, during a dysentery (*sekiri*) epidemic, he isolated and identified the causative organism (later named *Shigella dysenteriae*) using Koch's postulates, laying the foundation for enteric disease research. Shiga later worked with Paul Ehrlich in Germany, pioneering chemotherapy research for trypanosomiasis. Upon returning to Japan, he continued studying infectious diseases such as tuberculosis and beriberi, significantly contributing to public health improvements. As a professor at Keio University School of Medicine, Shiga advanced medical education in Japan and abroad. In his later years, he returned to his hometown near Sendai, living in harmony with nature. His gentle demeanor and scientific achievements earned him deep respect and affection from the local community. Shiga's funeral was marked by a long line of mourners, a testament to the impact he had on those around him. His life embodied the balance between scientific inquiry and humanitarian spirit, profoundly influencing the development of bacteriology and public health globally.

## Introduction and background

Kiyoshi Shiga (1871-1957), a pivotal figure in modern Japanese bacteriology, made groundbreaking contributions to the field of infectious diseases that continue to shape global public health practices today [1,2]. His discovery of *Shigella dysenteriae* in 1897, which caused bacillary dysentery, marked a significant milestone in the understanding and treatment of enteric diseases [1,2]. This achievement not only established Shiga as a prominent scientist but also positioned Japan as a key player in the international scientific community during a period of rapid modernization. As we navigate the challenges of the COVID-19 pandemic and face the ongoing threat of emerging infectious diseases, Shiga's work assumes renewed significance [3]. His meticulous approach to identifying and characterizing pathogens, as well as his emphasis on the practical application of scientific discoveries in public health, provides a valuable model for contemporary researchers and policymakers. The current global health crisis has underscored the critical importance of robust infectious disease research and the need for international collaboration in addressing pandemics - principles that are central to Shiga's career and philosophy. Shiga's legacy extends beyond his eponymous discoveries. His research on dysentery laid the groundwork for the development of modern approaches to enteric diseases, influencing the current strategies for diagnosis, treatment, and prevention. Moreover, his work alongside renowned scientists, such as Paul Ehrlich, and his mentorship under Shibasaburo Kitasato contributed to the global exchange of scientific knowledge, a practice that remains crucial in today's interconnected world [4].

In the context of increasing antibiotic resistance and the persistent threat of waterborne diseases in many parts of the world, Shiga's contribution to understanding bacterial pathogens and their transmission mechanisms remains highly relevant [5]. His work continues to inform strategies for managing the outbreaks of Shigella and other enteric pathogens that still pose significant public health challenges, particularly in developing countries. This paper explores Shiga's life, his significant contributions to bacteriology, and their enduring impact on contemporary medical practices and public health policies. As we face current and future global health challenges, revisiting the work of pioneers like Kiyoshi Shiga not only honors their contributions but also provides inspiration and guidance for addressing the complex health issues of our time.

## Review

Early life and education

Dr. Kiyoshi Shiga was born on February 7, 1871, in Sendai, Miyagi Prefecture, Japan, during a period of significant transformation in Japanese society, known as the Meiji Restoration. He was the fifth child of Shin and Chiyo Sato, a family of the Samurai class who experienced severe economic hardship following the collapse of Japan’s feudal system during the restoration. Due to these financial difficulties, Shiga was raised by his maternal family and later adopted its surname, Shiga, symbolizing a shift in his upbringing amidst the turbulent backdrop of Japan's industrialization and modernization [1,2].

Shiga excelled in mathematics during high school in Tokyo and entered the Tokyo Imperial University School of Medicine in 1892. He was inspired by Dr. Shibasaburo Kitasato, a prominent scientist known for his work on infectious diseases such as tetanus and diphtheria [4]. Kitasato, in collaboration with Emil Behring, demonstrated how immunity against these diseases could be developed in animals, a discovery that greatly influences immunology. Impressed by Kitasato’s approach, Shiga decided to pursue a career in microbiology under his mentorship [6].

Career beginnings and discovery of *Shigella dysenteriae*

After graduating in 1896, Shiga joined the Institute for Infectious Diseases in Tokyo, established and directed by Kitasato going on to make significant contributions to microbiology. Shiga initially focused on tuberculosis and diphtheria, but in 1897, his research took a pivotal turn when Japan was struck by a devastating outbreak of dysentery known locally as *sekiri*. The 1897 *sekiri* epidemic affected more than 91,000 people, with a mortality rate exceeding 20%. Dysentery is a poorly understood disease, characterized by violent diarrheal disturbances often accompanied by blood and mucus in the stool, severe abdominal pain, and tenesmus. Shiga expressed that this discovery "stirred my young heart with the hope of eradicating the disease and saving the suffering of about one hundred thousand cases that occurred yearly in my country at that time" [7]. 

To identify the cause of a deadly dysentery outbreak, Shiga used rigorous microbiological methods based on Koch’s postulates to isolate and characterize the responsible pathogen [8]. He analyzed samples from 36 patients and successfully isolated a gram-negative bacillus from their stool that fermented dextrose, was indole-negative, and did not produce acid from mannitol. To confirm its pathogenicity, Shiga showed that subcultures of the isolated bacillus could induce diarrhea in animal models, such as dogs.

**Figure 1:**
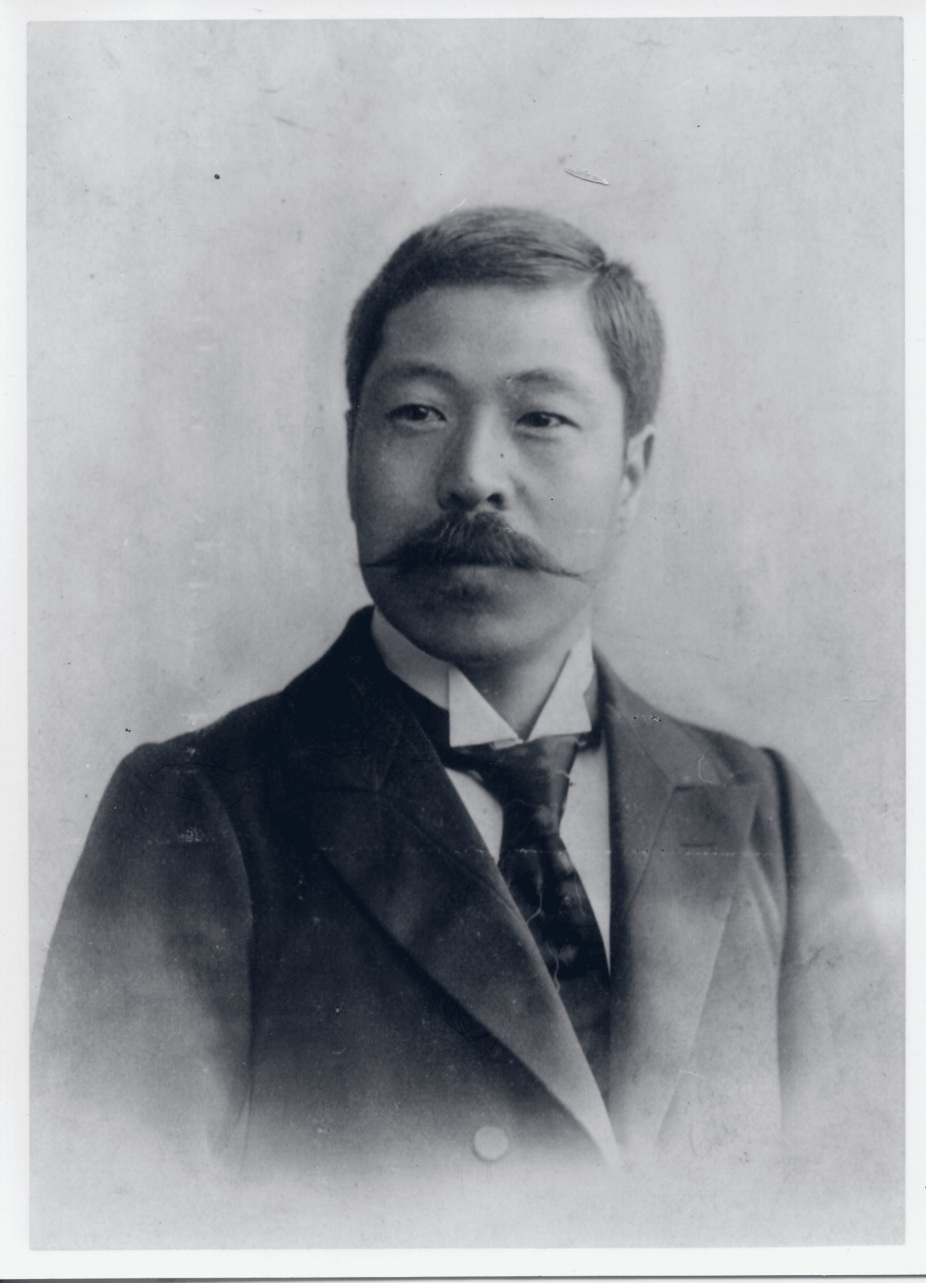
Dr. Kiyoshi Shiga, 31 years old Dr. Kiyoshi Shiga during his study abroad at the Ehrlich Institute in Germany. Photo courtesy of the Yamamoto Town Museum of History and Folklore. This image is in the public domain due to copyright expiration.

A critical part of Shiga’s discovery was his use of an agglutination technique, exposing the isolated organism to the serum of patients with convalescent dysentery, which caused the bacteria to coalesce, thus confirming its role as the causative agent. Initially named *Bacillus dysenteriae*, the organism was later renamed *Shigella dysenteriae*, representing one of the rare instances where a major pathogenic bacterium was named after a Japanese scientist. Shiga also identified a potent toxin produced by the bacterium, now known as Shiga toxin, which is significant in the pathogenesis [9-11]. Figure 1 shows Kiyoshi Shiga around the time he discovered the Shiga toxin.

International research and contributions to chemotherapy

Shiga went to Germany in 1901 and joined the laboratory of Paul Ehrlich at the Institut für Experimentelle Therapie (Institute for Experimental Therapy) in Frankfurt, a renowned research institute focused on pioneering chemotherapy methods. Ehrlich, a pioneer in the field of chemotherapy, was known for his innovative use of dyes to selectively target pathogens, which laid the foundation for modern therapeutic approaches. Shiga collaborated with Ehrlich to develop treatments for trypanosomiasis, a parasitic disease that is prevalent in Africa. Together, they discovered that Trypan Red, a dye effective against trypanosome parasites, represents one of the earliest successful applications of chemotherapy to treat parasitic infections [12].

Shiga’s work with Ehrlich significantly bolstered his reputation as an international authority on bacteriology. Upon his return to Japan in 1905, Shiga continued his research at the Kitasato Institute, contributing to the fields of bacteriology and immunology. His interests extended beyond dysentery to include other infectious diseases such as tuberculosis and beriberi, where he explored various therapeutic and preventive strategies. In 1914, Shiga joined Kitasato in founding the Kitasato Institute, an institution that remains the cornerstone of Japanese medical research today [4].

Two mentors

Kiyoshi Shiga's scientific journey was profoundly shaped by two influential mentors: Professor Paul Ehrlich and Professor Shibasaburo Kitasato. From Ehrlich, Shiga learned that implementing a scientific spirit and investigative enthusiasm required more than just manual techniques; it demanded mental skills and the ability to overcome research obstacles. Ehrlich emphasized the four "G"s of research: *Geld* (money), *Geduld *(patience), *Glück* (luck), and *Geschick* (skill). Shiga, recognizing that money was beyond his control and believing luck was on his side, focused on developing his skills through innate Japanese dexterity and cultivating patience to meet Ehrlich's expectations. Kitasato, on the other hand, left an indelible impression on Shiga during a lecture on plague bacillus cultivation at Tokyo University around 1894. Kitasato, dressed in traditional haori and hakama, displayed a dignified manner and was highly articulate, tinged with the Kumamoto accent, convincing Shiga that Kitasato would be his lifelong mentor. Despite the growing conflict between the university and the Institute for Infectious Diseases, Shiga joined the Kitasato Institute. He admired Kitasato's unwavering commitment to his decisions and his meticulous step-by-step approach to experimental work. Through the guidance of these two remarkable scientists, Shiga developed a robust scientific foundation that would serve him throughout his career, blending Ehrlich's emphasis on mental preparedness with Kitasato's precision and perseverance in research [2].

Academic leadership and public health advocacy

In 1920, Shiga became a professor at the Keio University School of Medicine, where he advanced medical education in Japan. Later, at the Japanese government’s request, he moved to Korea, then under Japanese rule, serving as the director of the National Hospital of Seoul and playing a key role in establishing Keijo University (now Seoul National University). He became the first Dean of the School of Medicine and later served as University President from 1929 to 1931. Shiga’s efforts significantly increased the standards of medical education and research in Korea. However, his tenure was marked by controversy, particularly his advocacy for controversial leprosy management measures, which led to his resignation in 1931. While his work in Korea is notably emphasized on platforms such as Wikipedia, it is less prominently featured on official sites such as the University of Tokyo, reflecting differing perspectives on his legacy.

Shiga’s contributions to public health have extended to the fight against tuberculosis. In 1924, Shiga acquired a strain of Bacillus Calmette-Guérin (BCG), known as BCG Tokyo 172 strain, from Albert Calmette in Paris. He brought this strain back to Japan, where it became the basis for developing Japan’s tuberculosis vaccine and tuberculin tests, which were pivotal to the country’s public health efforts against the disease. Throughout his career, Shiga was an advocate of preventive medicine and public health education. He participated in various public health campaigns, emphasizing the importance of hygiene and disease prevention, especially in China, where he contributed to tuberculosis education.

Final years and legacy

**Figure 2:**
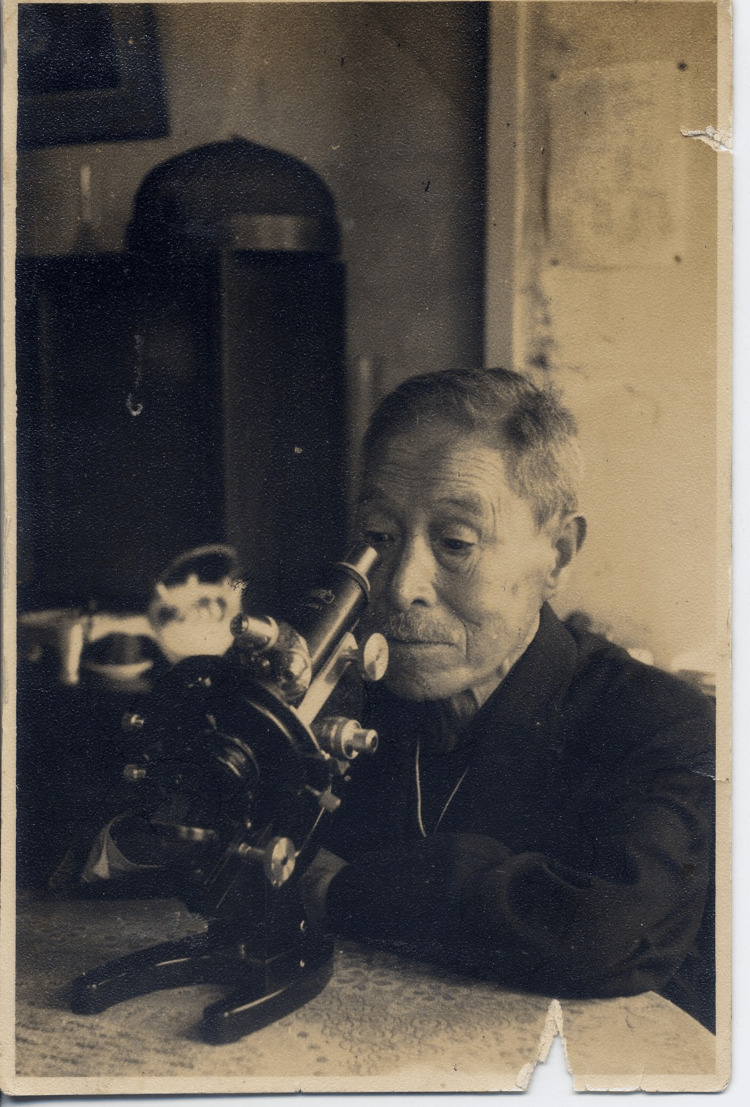
Dr. Kiyoshi Shiga, 77 years old Dr. Kiyoshi Shiga at the Kitasato Institute. Photo courtesy of the Yamamoto Town Museum of History and Folklore. This image is in the public domain due to copyright expiration.

Kiyoshi Shiga’s personal life was marked by both joy and profound loss. He married Ichiko in 1900 and had eight children. Despite his professional achievements, Shiga endured significant personal tragedies, including the death of his wife, Ichiko, from stomach cancer in 1944. Shortly after, his eldest son, Naoshi, a university professor in Taipei, was killed at sea while returning for his mother’s funeral, and another son, Akira, died from tuberculosis contracted during military service in China. Shiga's Tokyo home was destroyed during the World War II bombings. Figure 2 shows Kiyoshi Shiga at work even at an advanced age.

These hardships deeply affected Shiga, prompting him to retire to his villa, Kiyosuiso, in Isohama, near Sendai. In his final years, Shiga found solace in nature and reconnected with his roots, embodying the Japanese ideal of tranquility and renewal through simplicity. He continued to engage with the scientific community through writing and correspondence, completing the autobiography and biography of his mentor, Paul Ehrlich. Shiga cherished his retreat at Isohama as a sanctuary, reflecting his enduring resilience and pursuit of peace amid life's challenges.

Shiga's legacy extends beyond his scientific achievements; he was also recognized for his role as an international ambassador of goodwill during the tumultuous period of the Second Sino-Japanese War and World War II. He received numerous honors, including the Order of Culture in 1944 and Order of the Sacred Treasure, 1st class, awarded posthumously in 1957. His discovery of *Shigella dysenteriae* established the bacterial cause of dysentery and laid the foundation for future research on enteric diseases. His life exemplifies dedication, resilience, and the relentless pursuit of scientific progress, underscoring the importance of perseverance and humility.

Shiga's final reflections, shared during a Harvard University event, expressed both hope and disappointment at the persistence of dysentery: “The discovery of the dysentery bacillus stirred my young heart with hopes of eradicating the disease… The light of hope that once burned so brightly has faded as a dream of a summer night. This sacred fire must not die out.” His parting words, “Follow the mentor's spirit, not the mentor's footsteps,” capture the essence of his enduring influence, inspiring future generations to pursue knowledge with tenacity and integrity.

**Figure 3:**
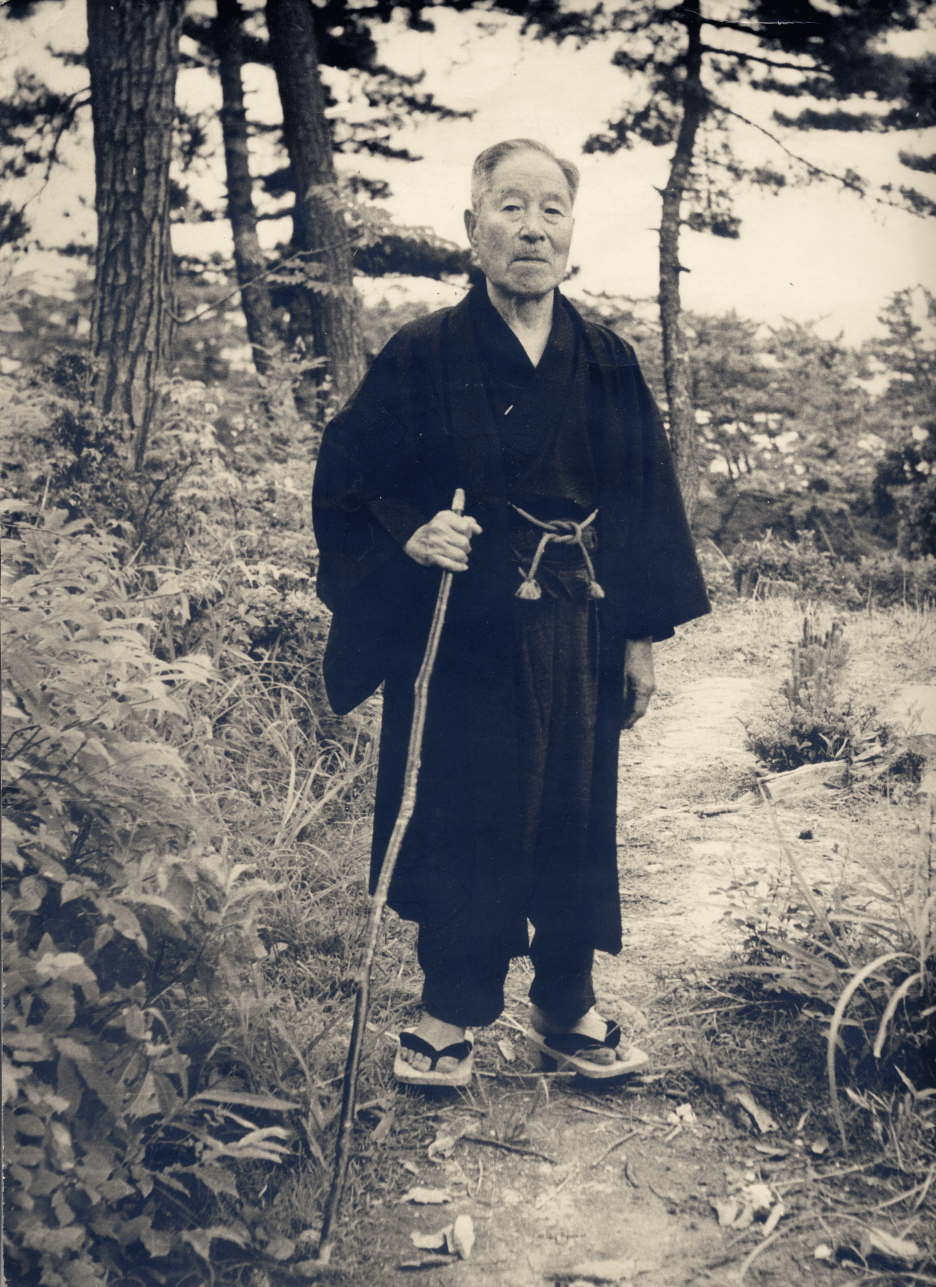
Dr. Kiyoshi Shiga, 81 years old Dr. Kiyoshi Shiga in the coastal pine forest near his villa in Isohama, Yamamoto Town. Photo courtesy of the Yamamoto Town Museum of History and Folklore. This image is in the public domain due to copyright expiration.

His dedication to public health and his role as an educator and leader were complemented by his gentle, humble nature, which endeared him to the local community. He was deeply cherished by those around him, and his funeral was marked by a long line of mourners - a testament to the respect and affection he inspired throughout his life [2]. Figure 3 shows Kiyoshi Shiga, who has been greatly respected and loved by many people, in his later years.

## Conclusions

Dr. Kiyoshi Shiga left behind not only outstanding achievements as a bacteriologist but also lived a life rich in humanity, overcoming personal tragedies. In his later years, he continued to embody scientific passion and humility, while living in harmony with nature. His discovery of *Shigella bacillus* and its contributions to public health continue to have a profound impact on modern infectious disease research and countermeasures.
